# Record-low coastal sea levels in the Northeast Pacific during the winter of 2013–2014

**DOI:** 10.1038/s41598-019-40397-w

**Published:** 2019-03-07

**Authors:** Yaqi Wang, Hailong Liu, Pengfei Lin, Jianjun Yin

**Affiliations:** 1 State Key Laboratory of Numerical Modeling for Atmospheric Sciences and Geophysical Fluid Dynamics, Institute of Atmospheric Physics, Chinese Academy of Sciences, Beijing, 100029 China; 2 College of Earth Sciences, University of Chinese Academy of Sciences, Beijing, 100049 China; 3 Department of Geosciences, University of Arizona, Tucson, AZ 85721 USA

## Abstract

During the winter of 2013–2014, the averaged tide gauge (TG) coastal sea level (CSL) anomaly north of 40°N was a record low of −107 mm for the period of 1948–2016. Statistical analysis indicates that this large drop was a once-in-a-century event and closely related to an unusual ocean warming event known as “The Blob”. The Blob developed in the NE Pacific during the winter of 2013–2014. Both the Blob and record-low CSL can be attributed to wind changes associated with an unusually high sea level pressure (SLP) pattern over the NE Pacific. The anomalous local longshore winds induced by the positive SLP anomalies caused strong offshore Ekman transport along the coast of NE Pacific, thereby leading to the record-low CSL. In addition, the steric sea level changes also contributed a significant part (17%) to the record-low CSL. The Pacific Decadal Oscillation (PDO), as the primary variability mode in the NE Pacific on decadal time scales, did not contribute to the emergence of this extreme CSL event.

## Introduction

**Figure 1:**
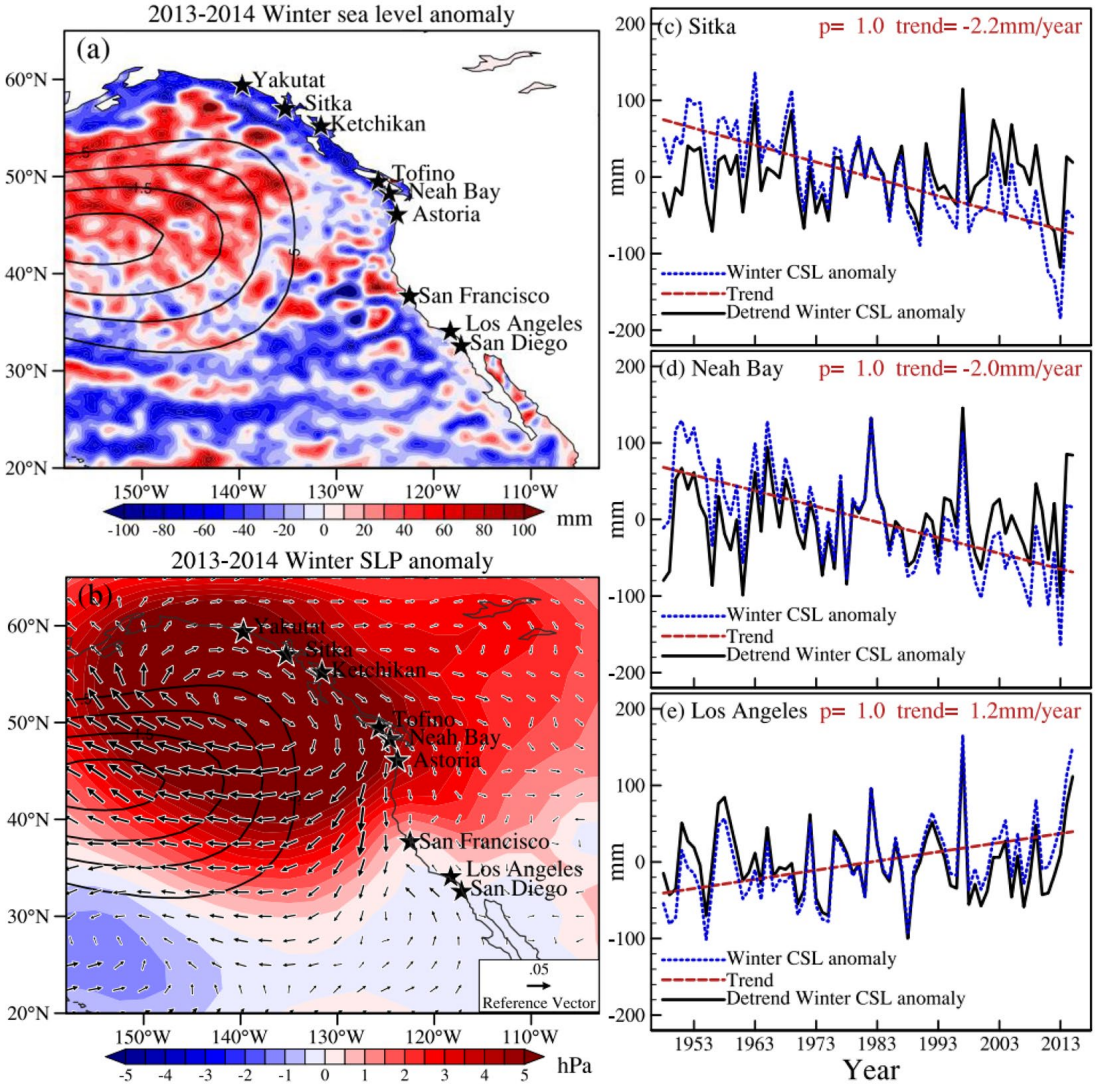
**(a)** AVISO sea level anomaly (mm; shading) for the winter of 2013–2014. **(b)** Anomalies of SLP (hPa; shading) and wind stress (N/m^2^; vector) for the winter of 2013–2014. The black stars in **(a**,**b)** represent the locations of TG stations. Contours in **(a)** and **(b)** represent the SST anomalies greater than 0.5 °C from ERSST for the winter of 2013–2014. The winter CSL anomaly (blue lines) for **(c)** Sitka, **(d)** Neah Bay and **(e)** Los Angeles for the period 1948–2016. Linear trends of winter CSL anomaly (red lines) and the detrended time series (black lines) are also shown in **(c**–**e)**.

Coastal sea levels (CSL) manifest important variability and changes of the coupled ocean-atmosphere system. Here we focus on the sea level change along the coast of the Northeast (NE) Pacific. In this region, CSL shows a near-zero or even decreasing trend over the past three decades, in sharp contrast to the global mean sea level rise^1–4^. Previous studies have indicated that the CSL trend in the NE Pacific is related to shifts in wind patterns associated with the Pacific Decadal Oscillation (PDO)^3–6^. While many coastal regions worldwide recently experienced record-high CSL, record-low CSL was reached in the NE Pacific during the winter of 2013–2014 (Fig. 1a, color), likely related to the so-called “Blob”. The Blob refers to the unusually warm sea surface temperature (SST) in the NE Pacific in winter 2013–2014 with the largest anomalies of more than 2 °C (Fig. 1a, contour). Bond *et al*.^7^ pointed out that the development of the extraordinarily warm SST anomalies in the NE Pacific can be attributed to anomalously weak surface winds induced by the strong positive sea level pressure (SLP) anomalies (Fig. 1b, color), which suppressed the local ocean heat loss to the atmosphere and reduced horizontal advection and vertical mixing in the upper ocean. This extreme warm SST event and its effects on marine life have aroused widespread concerns^8–10^, as the NE Pacific ecosystems showed significant changes following the arrival of the Blob^9^. In contrast to ocean temperature, sea level signals associated with this significant event have not been investigated yet. Given that their relationship is established, sea level data could be used to quantify how extreme the Blob was in the NE Pacific.

The longshore component of the anomalous wind pattern induced by SLP anomalies is usually considered to be one of the main factors to influence CSL^6,11^. Sturges and Douglas^12^ pointed out that longshore wind stress is one of the primary mechanisms for causing CSL change on the eastern side of the ocean and used longshore wind stress to discuss its effects on estimates of CSL rise. Bromirski *et al*.^4^ showed that interdecadal trends in PDO-related wind stress curl can account for the suppression of CSL trends in the NE Pacific from 1980 to 2010. Moreover, they warned that an imminent phase shift in the PDO-related wind stress could lead to a sharp increase in NE Pacific CSL in the coming years. Thompson *et al*.^6^ used the method of multiple linear regression to examine the relative contributions from remote equatorial wind stress, local longshore wind stress, and local wind stress curl, and suggested that the decreased rate of sea level rise along the coast of NE Pacific from 1992–2010 is primarily due to the ocean’s response to the strengthened trade winds, with longshore wind stress becoming important to CSL trends from 37°N to 48°N. Most of these studies were concerned about the CSL trends in the NE Pacific over the past three decades, but extreme year-to-year changes have received less attention. The study of extreme CSL rise and fall is related to practical aspects of coastal zone threats and activities and allows us to identify important warning levels for floods and coastal protection services as well as shipping safety^13,14^. It is worth noting that the maximum value of the 2013–2014 SLP anomaly is located adjacent to the coast of NE Pacific (Fig. 1a). Therefore, it is reasonable to presume that highly anomalous CSL will appear during the winter of 2013–2014.

Here we focus on the winter CSL anomalies along the Pacific coast of North America from California to Southeast Alaska. We first identify and quantify the record-low CSL during the winter of 2013–2014. Then, the cause for this extreme CSL anomaly and the role of large-scale climate modes are investigated.

## Results

### Observed record-low CSL in the NE Pacific

Following Bond *et al*.^7^, we use four-month (October-January) mean values to represent winter anomalies along the coast of the NE Pacific. Figure 1a shows the AVISO sea level anomaly during the winter of 2013–2014 (October 2013-January 2014). Along the coast of western North America, the CSL anomaly north of 40°N is lower than it is to the south. This coincides with the position of the positive SLP anomaly, located north of 40°N (Fig. 1b). We select nine TG stations with continuous and relatively long data with high-quality: San Diego, Los Angeles, San Francisco, Astoria, Neah Bay, Tofino, Ketchikan, Sitka and Yakutat. All have records during the period of 1948–2016. TG winter CSL anomalies are calculated relative to the winter mean from 1948 to 2016. Figure 1c–e shows the winter CSL anomaly (blue line) from three representative TG stations at different latitudes: Los Angeles (33.7°N) for positive CSL anomaly, Sitka (57.1°N) for extreme negative CSL anomaly, and Neah Bay (48.4°N) in between. The CSL anomalies at both Neah Bay and Sitka show the lowest values during the winter of 2013–2014, −163 mm for Neah Bay and −185 mm for Sitka, superimposed on significant negative trends during the period of 1948–2016, −2.2 mm/year and −2.0 mm/year, respectively. The negative values indicate domination of local vertical land motion (VLM) over the long-term global mean sea level trend. Even after removing these linear trends, the CSL anomaly is still the lowest in the winter of 2013–2014 (−99 mm for Neah Bay and −118 mm for Sitka; black line). In contrast, Los Angeles shows a positive trend of CSL during the period of 1948–2016 (1.1 mm/year) and the value of winter 2013–2014 is not the lowest both with and without the trend. The observed trends of winter CSL anomalies at the nine TG stations for two periods in 1948–2016 and 1993–2016 are provided in Table S1. VLM is estimated based on the difference between absolute sea level (ASL; AVISO) and relative sea level (RSL; TG) over the period of 1993–2016^15^. The decreasing trend of TG RSL is largely due to land uplift for the period of 1993–2016. In the present study, we estimate VLM based on the data after 1993. The trend of VLM may be different from 1948 to 2016. To gain insight into the cause of the 2013–2014 record-low CSL event in the NE Pacific, we mainly focus on the variables with the linear trends removed.

**Figure 2:**
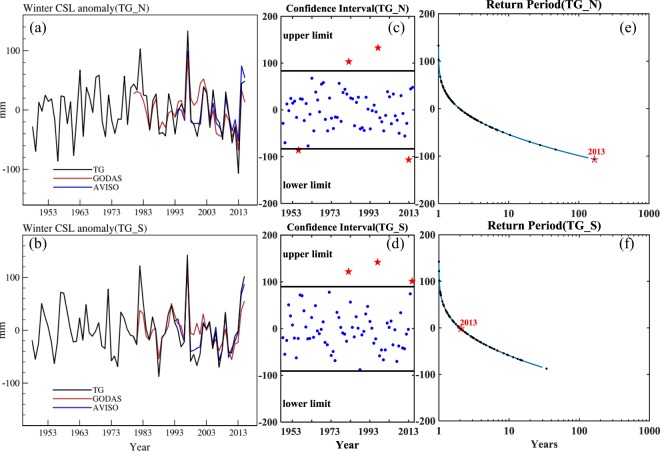
Composite of **(a)** TG_N and **(b)** TG_S winter CSL anomalies in the NE Pacific for three datasets, black for TG, red for GODAS and blue for AVISO, respectively. The data from AVISO and GODAS are area-averaged over 1° × 1° boxes at the TG stations. **(c**,**d)** are the 95% confidence intervals corresponding to Gaussian distribution of winter CSL anomalies in the two regions. **(e**,**f)** are the return period for the two regions.

To further verify the emergence of the record-low CSL for the winter 2013–2014, we divide the coastal TG stations into two regions: north and south of 40°N, represented as TG_N and TG_S. The TG_N time series is calculated as the mean of six stations between Astoria and Yakutat and the TG_S time series is averaged of three stations for the south (San Diego, Los Angeles and San Francisco) (Fig. 2a,b). In addition to the detrended TG data, the detrended satellite data from AVISO and the detrended assimilation data from GODAS area-averaged over 1° × 1° boxes at the TG stations are also used to confirm the record-low CSL in the NE Pacific. Winter CSL anomalies for AVISO and GODAS are constructed by removing the winter mean computed over their corresponding period (AVISO in 1993–2016 and GODAS in 1980–2016). All three datasets show the lowest CSL anomalies during the winter of 2013–2014 (Fig. 2a). The most pronounced CSL drop associated with this event can be found in the TG data, in which the TG_N CSL anomaly decreased to −107 mm, much larger than that for AVISO (−52 mm) and GODAS (−68 mm). The difference of the three datasets can be due to the fact that the TG data is a point measurement at the coast while AVISO and GODAS gridded data are area averaged near the coast and outside the primary zone of Ekman transport convergence that peaks along the coastline. At the same time, we notice that the two maxima of winter CSL anomalies in Fig. 2a,b correspond to strong El Niño years (1982 and 1997), indicating the CSL anomaly in the NE Pacific may also reflect remotely-forced oceanic signals from the tropics^16^.

The 95% confidence intervals corresponding to the Gaussian distribution of the winter CSL anomalies over the period of 1948–2016 in the two regions are shown in Fig. 2c,d. For the winter of 2013–2014, the TG_N value is below the low limit of the confidence interval and the TG_S value is in the confidence interval. The 1982 and 1997 values are located above the upper limit of the confidence interval in both Fig. 2c,d, indicating CSL extremes during those two strong El Niño events. Except for the record-low value of winter 2013–2014, the value of winter 1955–1956 is also lower than the confidence interval, but not as extreme as that of 2013–2014. Furthermore, we fit the Gaussian distribution to the winter CSL anomalies in the two regions from 1948 to 2016 to calculate the return period. In Fig. 2c, the red star represents the regression return period corresponding to the value of winter 2013–2014. The result in Fig. 2e shows that the TG_N CSL anomaly for the winter of 2013–2014 is rare and a once-in-a-century event. For the TG_S CSL anomaly, the return period is less than 5 years suggesting a common event. Combining Fig. 2a,c,e, we find that the TG_N CSL anomaly in the winter of 2013–2014 was the lowest event on record from 1948–2016.

### Causes of the record-low CSL

The variability of CSL south of San Francisco is dominated by coastally trapped waves of tropical origin, while local wind stress is increasingly important to the north^6^. We find no record-low CSL value south of 40°N during winter 2013–2014 (Fig. 2b), suggesting that the signal of the extreme event is not from the tropics through coastally-trapped waves, but rather from the local wind stress forcing. Following Thompson *et al*.^6^, we use a multiple linear regression model to compare the contributions of local longshore wind stress (${{\rm{\tau }}}_{{ls}}$), local wind stress curl (${{\rm{\tau }}}_{{xy}}$) and remote equatorial wind stress (${{\rm{\tau }}}_{{eq}}$) to the variability of TG_N winter CSL. ${{\rm{\tau }}}_{{eq}}$ is computed as the average zonal wind stress over the region spanning 6°N–6°S and 150°–280°E, ${{\rm{\tau }}}_{{xy}}$ is calculated area-averaged over 5° × 5° boxes at the TG_N stations. ${{\rm{\tau }}}_{{ls}}$ is computed by interpolating the vector wind stresses to the TG_N stations and projecting onto an approximate shoreline angle within a 2° radius of each TG_N station. The normalized time series of winter anomalies and the wind stress time series scaled by their respective regression coefficients are shown in Fig. S1. For the long-term time series, the result is consistent with Thompson *et al*.^6^ that most of the variance of the regression results can be explained by ${{\rm{\tau }}}_{{eq}}$ compared to ${{\rm{\tau }}}_{{ls}}$ and ${{\rm{\tau }}}_{{xy}}$. For the winter of 2013–2014, it can be mostly explained by the local longshore wind stress. The local longshore wind stress anomalies associated with the SLP anomaly in the NE Pacific (Fig. 1a) drives offshore Ekman transport, and thus lower CSL anomaly.

**Figure 3:**
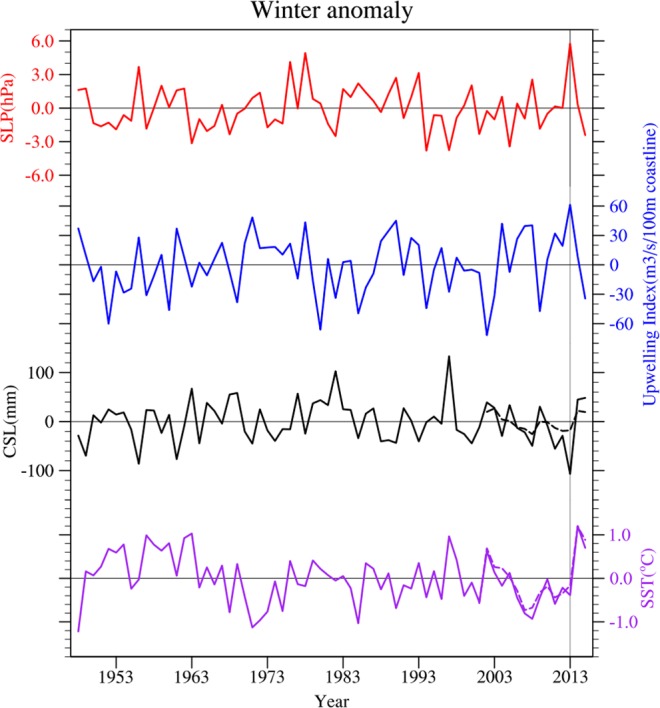
The TG_N station averaged winter anomalies for SLP (red, NCAR-NCEP), upwelling index (blue, PFEL), CSL (black-solid, TG) and SST (purple-solid, NCAR-NCEP) during the period of 1948–2016. The winter SLP and SST anomalies are calculated here by using the grid value averaged over 5° × 5° boxes at the TG_N stations. The winter upwelling index is taken from the average of 6 locations from 42°N to 57°N. The Argo winter SST (purple-dashed) and calculated winter SSL anomaly (black-dashed; minus global mean SSL) averaged over 4° × 4° boxes at the TG_N stations are also shown by removing the monthly anomalies and linear trends during the period of 2002–2016.

To understand the cause of the record-low CSL in the NE Pacific, the winter anomalies of SLP, upwelling index, CSL and SST spatially averaged for the TG_N stations are plotted in Fig. 3. The linear trends are also removed in each time series. During the winter of 2013–2014, the TG_N SLP anomaly is the highest during the analysis period (Fig. 3, red line). It drives an anticyclonic surface wind anomaly pattern over the NE Pacific. The local longshore wind stress anomalies produce offshore Ekman mass transport and cause strong coastal upwelling anomalies from southeast Alaska to northern California (Fig. 3, blue line). Therefore, the TG_N CSL reaches its lowest value during the winter of 2013–2014 (Fig. 3, black line). The correlation coefficients between TG_N CSL and the other three all reach their respective maximums at zero-lag and the correlation coefficient between CSL and SLP/upwelling index is significant (>0.6). We also find that the TG_N anomalies of SLP and upwelling index in the winter of 2013–2014 are also extreme values, further supporting the mechanism described above.

In addition, the stronger upwelling (i.e., positive anomalies) also leads to negative SST anomalies (Fig. 3, blue line) and a decrease in seawater mass loading on the shelf along the west coast of North America. Stronger upwelling can bring cooler subsurface waters to the surface, thereby resulting in a negative steric sea level (SSL) anomaly due to seawater density changes. To evaluate the contribution of the SSL anomaly to the TG_N record-low CSL, we use gridded Argo temperature and salinity data to calculate the local SSL anomaly through equation (1) listed in the Methods section for the period of 2002–2016. Compared with the ERSST long term SST anomaly time series (Fig. 3, purple-solid line), the Argo winter SST anomaly time series (Fig. 3, purple-dashed line) depicts most of its variability. The calculated TG_N SSL anomaly (Fig. 3, black-dashed line) for the winter of 2013–2014 is −18 mm, which accounts for about 17% of the total sea level drop (−107 mm). Thus, the steric effect is an important factor. In the open ocean, the warmer seawater in the blob region could cause a 20 mm rise of SSL (Fig. S2d, red-dashed line), which explains most of the open ocean sea level anomaly (26 mm; Fig. S2d, black line). This is consistent with the previous study by Wu *et al*.^17^, which found that in the northeast subtropical Pacific, the mass component is critical for the variability of CSL, while the steric effect dominates the open ocean sea level changes.

### Role of large-scale climate modes

**Figure 4:**
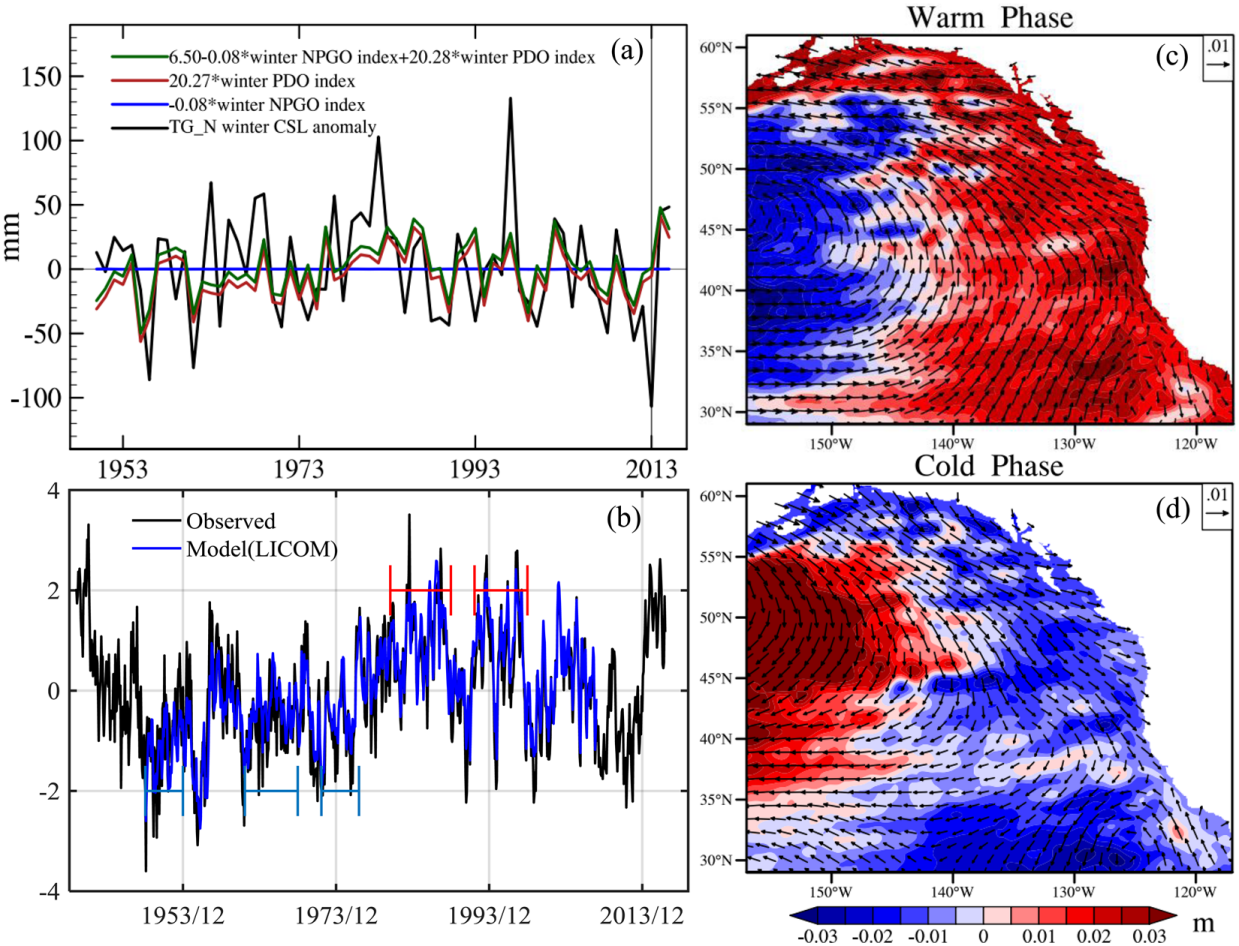
(**a**) TG_N winter CSL anomaly (black) and the result of a multiple linear regression onto winter PDO index(red) and winter NPGO index(blue). **(b)** The PDO index for the model (blue, LICOM) and the observation (black, JISAO). The warm and cold periods we choose are shown by the red and blue lines, respectively. The specific cold years are 1949–1953, 1962–1968 and 1972–1976, the warm years are 1981–1988 and 1992–1997. The composite of annual mean sea surface height (m; shaded) and surface wind stress (N/m^2^; vector) anomalies during **(c)** the cold and **(d)** the warm phase of the PDO.

CSL changes have been linked to large-scale climate variations in the NE Pacific such as the PDO^10,18,19^, North Pacific Gyre Oscillation (NPGO)^10,20^ and El Niño^16,21^, and only the Kelvin waves associated with strong El Niño events typically affect CSL changes poleward of San Francisco^22^. For the winter of 2013–2014, the absence of a strong ENSO event precludes the possible role of coastal wave propagation in the extreme CSL event. We therefore focus on the role of PDO and NPGO. In Fig. S3, we show the normalized time series of winter PDO index (Fig. S3, red-dashed line), NPGO index (Fig. S3, blue-dashed line) and TG_N CSL anomaly (Fig. S3, black line), respectively. The correlation coefficient between TG_N CSL anomaly and PDO (0.48) is much larger than that between CSL anomaly and NPGO (−0.03), indicating that the variability of the winter CSL north of California is dominated by PDO rather than NPGO. This result is consistent with the studies of Di Lorenzo *et al*.^20^ and Chhak *et al*.^23^, which pointed out the coastal upwelling and offshore volume/mass transport north of about 40°N is dominated by the PDO, whereas the NPGO dominates south of this latitude band along the California Current System. To estimate the correspondence between the NPGO/PDO and the TG_N record-low CSL, we use a multiple linear regression model to regress the anomaly time series of TG_N winter CSL onto winter PDO index and winter NPGO index. The results of the multiple linear regression and the regression coefficients are shown in Fig. 4a. For the winter of 2013–2014, the TG_N record-low CSL is −107 mm (Fig. 4a, black line), with slightly negative contributions from PDO and NPGO (1 mm; Fig. 4a, green line). The part that can be explained by the NPGO is negligible for the entire period (Fig. 4a, blue line), therefore confirming the TG_N CSL is not dominated by the NPGO. Similarly, the variability induced by the PDO is insignificant (−5 mm; Fig. 4a, red line), indicating the PDO did not play a role in this extreme event during 2013–2014. However, the PDO is more important in the winter of 2014–15 (Fig. 4a, red line). This is in line with the assessment by Di Lorenzo and Mantua^10^ about the domination of PDO in the winter of 2014–15. This result is also consistent with the observed PDO index (Fig. 4b, black line) for the two winters.

It is worth noting that the observed PDO index in December 2013 is near zero, indicating that the winter of 2013–2014 is at the turning point of PDO from its negative (cold) to positive (warm) phase. To put this extreme event into context and further investigate the relationship between PDO and the CSL, we analyze the hindcast simulation of an eddy-resolving ocean model - LICOM. Figure 4b shows the comparison of the PDO index between observations (Fig. 4b, black line) and the model simulation (Fig. 4b, blue line). The correlation coefficient is significantly high (r = 0.90, P = 1.00). It indicates that the LICOM simulations can reproduce the large-scale climate modes reasonably well. The ensemble means of simulated sea level and wind stress anomalies for the cold phase shows negative CSL anomaly in the NE pacific (Fig. 4c, color), which is associated with anomalous southward longshore wind stress (Fig. 4c, vector). In the warm phase, we find positive CSL anomalies (Fig. 4d, color) and anomalous northward wind stress along the coast of NE Pacific (Fig. 4d, vector).

The winter of 2013–2014 is at the turning point of the PDO shift from its cold to warm phase, and from the lower to higher CSL in the NE Pacific. Although the PDO did not directly contribute to the emergence of this extreme event for the winter of 2013–2014, the subsequent shift of PDO to its warm phase (Fig. 4b, black line) can raise the 2014–2015 winter CSL in the NE Pacific (Fig. 4a, red line), thus making the CSL in the winter of 2013–2014 the lowest. Consistent with observations that show an intensification of the Aleutian Low in the NE Pacific during the winter of 2014–2015 (Fig. S4).

## Discussion and Conclusions

We find that large-scale climate modes did not contribute to the emergence of the record-low CSL in the NE Pacific in winter 2013–2014. However, the NPGO, El Niño and PDO can be linked by the evolution of the positive SLP anomaly over the NE Pacific for the winter of 2013–2014 and the following two winters^10,24–27^. The coastal SST anomaly is higher than normal in the winter of 2014–15 (Fig. 3, green line), corresponding to the 2014–15 NE Pacific marine heatwave^11^.

In the present study, we have investigated the winter CSL change in the NE Pacific during the period of 1948–2016 by using the TG, AVISO and GODAS datasets. We find that a record-low sea level event occurred during the winter of 2013–2014. The averaged TG CSL anomaly from southeast Alaska to northern California was −107 mm, which is a once-in-a-century event. The extreme event of CSL change is coincident with the occurrence of the Blob in the NE Pacific. Both phenomena are linked to a high atmospheric pressure system located near the NE Pacific coast. The higher-than-normal SLP led to an anomalous local longshore wind stress and thus caused the anomalous offshore Ekman transport. In addition, SSL change contributed about 17% of the total extreme. These ocean-atmospheric processes were responsible for the record-low CSL in the winter of 2013–2014, in sharp contrast to the near record-high globally averaged CSL during this same winter (https://www.climate.gov/news-features/understanding-climate/climate-change-global-sea-level).

## Methods

### Sea level datasets

We analyze three datasets to identify the variability of wintertime sea level along the coast of NE Pacific. Monthly mean tide gauge (TG) revised local reference dataset for 1948–2016 is obtained from the Permanent Service for Mean Sea Level (PSMSL) website (http://www.psmsl.org). The sea level measured by the tide gauge is a height to the level of benchmarks on the nearby land (Relative sea level; RSL), so vertical land motion will influence sea level change^28^. The inverted barometer (IB) effect on sea level is removed from the TG data using the NCEP/NCAR Reanalysis version 1 dataset. Second is the quarter-degree multiple altimeter data product, sea level anomaly, produced by Archiving, Validation, and Interpretation of Satellite Oceanographic (AVISO) covering the period from 1993 to 2016 (www.aviso.oceanobs.com). All classical geophysical and environmental corrections are applied, including the IB effect. Global mean sea level rise is removed for the period of 1993–2016. The sea level measured by AVISO is absolute sea level (ASL), vertical land motions (VLM) can be estimated based on the difference between ASL and RSL over the period of 1993–2016^15^. The third is dynamic sea level (sea surface height relative to geoid; DSL) from the NCEP Global Ocean Data Assimilation System (GODAS) datasets during the period of 1980–2016 (http://www.esrl.noaa.gov/psd/data/gridded/data.godas.html). This is ocean reanalysis data and does not include atmospheric pressure forcing, so the IB adjustment is not needed. Anomalies for the above datasets are constructed by removing the monthly climatology computed over their corresponding period (TG in 1948–2016, GODAS in 1980–2016 and AVISO in 1993–2016). Linear trends for the above datasets are removed over their corresponding period.

### Sea surface temperature (SST), sea level pressure (SLP) and wind stress datasets

To reconstruct the ocean and atmospheric variability associated with the NE Pacific winter anomalies we used NOAA extended reconstructed sea surface temperature (ERSST) V4 data, monthly mean SLP and wind stress data are obtained from NCEP/NCAR Reanalysis. We analyze the data over the period to 1948–2016. Anomalies for SST, SLP and wind stress are constructed by removing the monthly climatology over the period 1948–2016. The linear trends are removed from the anomalies at each grid point.

### Return period

The return period is used to show how extreme is the NE Pacific coastal sea level (CSL) during the winter of 2013–2014. The return period (T) is calculated as T = 1/Pr, where Pr is the cumulative probability density function associated with the Gaussian distribution of TG winter sea level. Previous studies adopted the Gumbel distribution when calculating the return period of extreme sea level events^14,15^. Here we find that the winter CSL in the NE Pacific is better fitted to the Gaussian distribution than the Gumbel distribution (Fig. S5).

### Steric sea level

Sea level change can be divided into steric and mass-induced components^29,30^. We use the gridded monthly temperature and salinity data from Argo for the period of 2002–2016 to further evaluate the contribution of steric effect to the winter sea level along the coast of NE Pacific, the data are available at the JAMSTEC Argo Web site (http://www.jamstec.go.jp/ARGO/J_ARGOe.html).

By neglecting nonlinear terms, the SSL is calculated as

1 $${\rm{SSL}}=\,-{\int }_{{Z}_{1}}^{{Z}_{2}}\frac{\rho (T,S)-\rho ({T}_{0},{S}_{0})}{\rho ({T}_{0},{S}_{0})}dz\,$$

where ${\rm{\rho }}$, *T* and *S* denote density, temperature, and salinity, respectively. We set ${z}_{1}$ to the depth of the 2000dbar and ${z}_{2}$ to the sea surface. *T*_0_ and *S*_0_ denote the temperature and salinity at the first-time step, respectively^17^. Anomalies are constructed by removing the monthly climatology over the period 2002–2016. The linear trends are removed from the anomalies at each grid point.

### PDO, NPGO and coastal upwelling index

The monthly PDO index (1948–2016) is obtained from the Joint Institute for the Study of the Atmosphere and Ocean (JISAO) at the University of Washington (http://jisao.washington.edu/pdo/PDO.latest). The observed PDO Index is defined as the leading principal component of North Pacific (north of 20°N) monthly SST variability^19^. The NPGO index (1950–2016; http://www.o3d.org/npgo) is defined as the second principal component of sea surface height anomalies in the NE Pacific (180°–110°W, 25°N–62°N)^20^. To show the intensity of offshore Ekman transports along the NE Pacific coast, the coastal upwelling index for six locations at each 3 degrees of latitude from 42°N to 57 N°(1948–2016) is taken from Pacific Fisheries Environmental Laboratory(PFEL; http://www.pfel.noaa.gov/products/PFEL/modeled/indices/upwelling/upwelling.html).

### LICOM model datasets

We use the hind-cast dataset from LASG/IAP Climate System Ocean Model version 2 (LICOM 2.0) to reconstruct the ensemble mean of sea level and wind stress anomalies associated with the cold and warm phase of the PDO. LICOM 2.0 was a global ocean general circulation model developed at the State Key Laboratory of Numerical Modeling for Atmospheric Sciences and Geophysical Fluid Dynamics of the Institute of Atmospheric Physics (LASG/IAP). The performance of the high resolution LICOM2.0 has been evaluated by Liu *et al*.^31^. The dataset has a horizontal resolution of 0.1° × 0.1° in longitudinal and latitudinal directions, respectively, and 55 vertical levels spanning the period from January 1949 to December 2007. The forcing dataset and algorithm are both from Coordinated Ocean-Ice Reference Experiments (COREs)^32^. Owing to the lack of a sea ice module in LICOM2.0, the sea ice concentration was determined by the observation dataset from Hadley Center, HadISST(https://climatedataguide.ucar.edu/climate-data/sea-ice-concentration-data-hadisst). The model PDO Index is calculated the same as the observed PDO Index for the period of 1949–2007.

## Supplementary information

Supplementary Information
