# Supplementary material for: Record-low coastal sea levels in the Northeast Pacific during the winter of 2013–2014
Source: Sci Rep. 2019 Mar 7;9:3774. doi: 10.1038/s41598-019-40397-w (PMC6405740; doi:10.1038/s41598-019-40397-w)
Supplement: Supplementary file 1 — Supplementary Information [file 41598_2019_40397_MOESM1_ESM.docx]

Supplementary Information:

**Record-low coastal sea levels** **in the Northeast Pacific**

**during the winter of 2013-2014**

**Yaqi Wang^1,2^, Hailong Liu^1, 2*^, Pengfei Lin^1,2^ & Jianjun Yin^3^**

^1^State Key Laboratory of Numerical Modeling for Atmospheric Sciences and Geophysical Fluid Dynamics, Institute of Atmospheric Physics, Chinese Academy of Sciences, Beijing 100029, China

^2^College of Earth Sciences, University of Chinese Academy of Sciences, Beijing 100049, China

^3^Department of Geosciences, University of Arizona, Tucson, AZ 85721, USA.

*Corresponding author: Hailong Liu ([lhl@lasg.iap.ac.cn](mailto:lhl@lasg.iap.ac.cn))

**
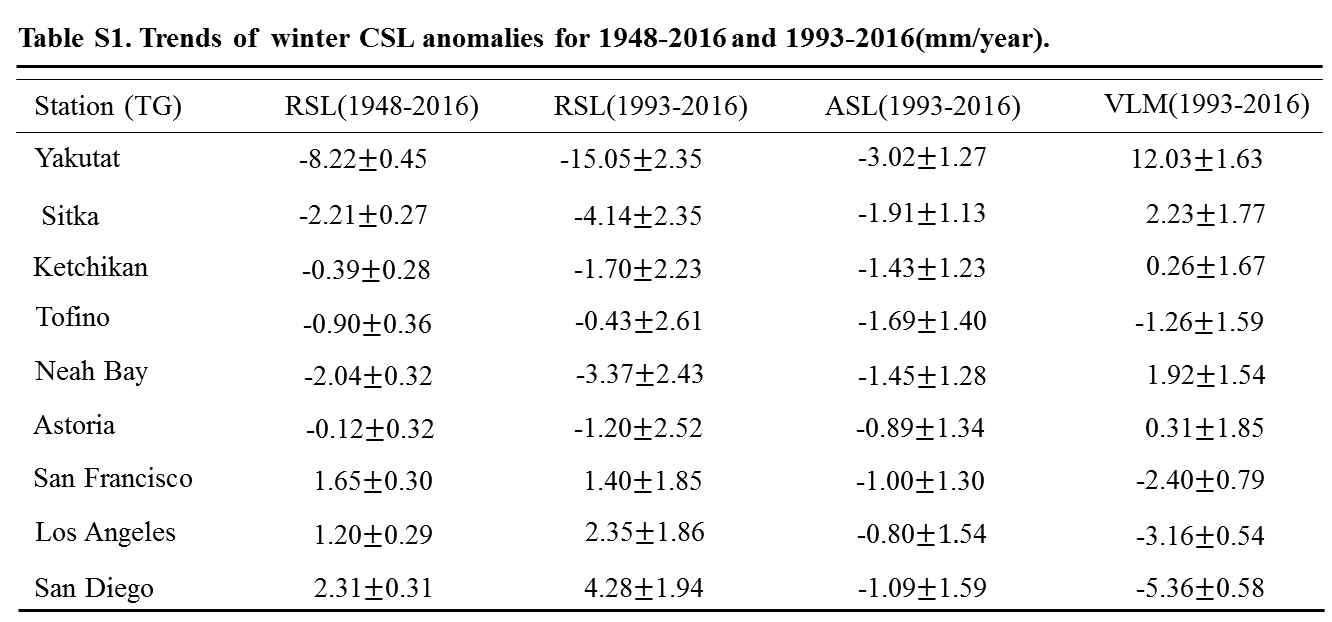
**


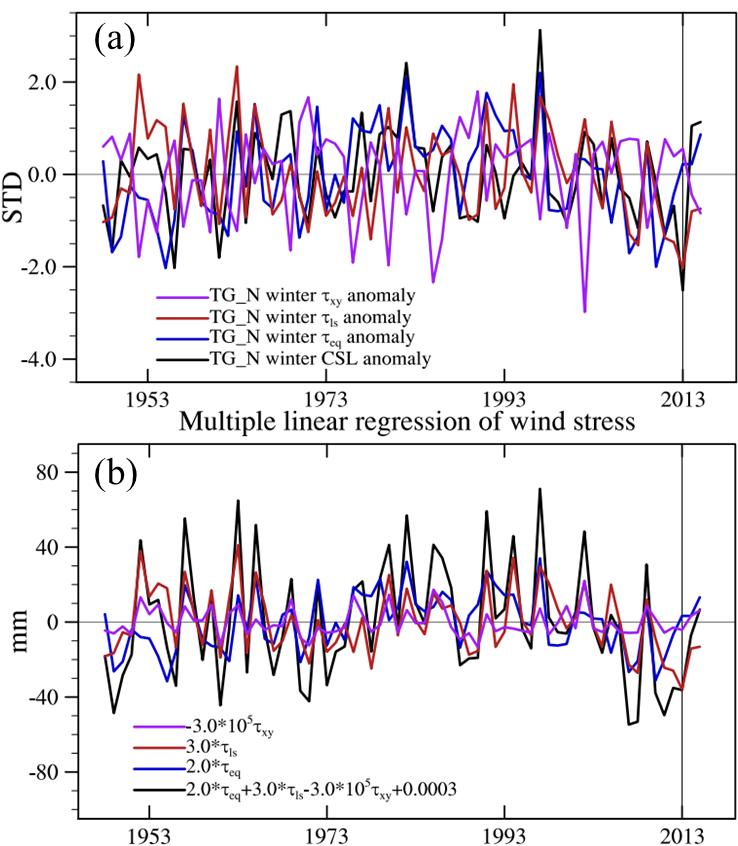


**Figure S1.** **(a)** Standardized time series for TG_N winter anomalies of $\tau_{eq}$ (blue line), $\tau_{ls}$ (red line), $\tau_{xy}$ (purple line) and CSL (black line), respectively. **(b)** The result of a multiple linear regression onto $\tau_{eq}$,$\tau_{ls}$ and $\tau_{xy}$.


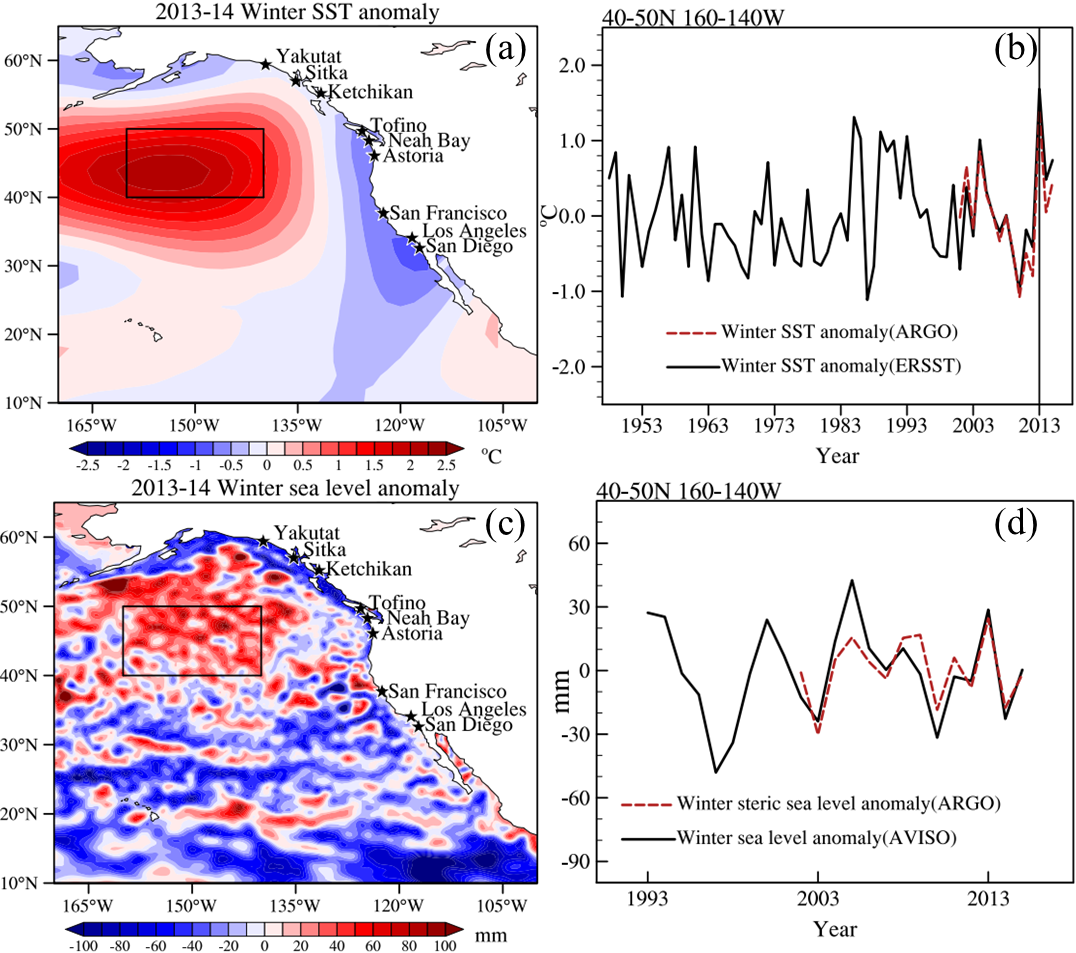


**Figure S2. (a)** ERSST SST anomaly for the winter of 2013-2014. **(b)** Winter SST anomaly from ERSST and ARGO over the area of 50 to 40°N, 160 to 140°W (indicated by the box shown in Figure S2a and S2c) for the period of 1948-2016 and 2001-2016, respectively. **(c)** AVISO sea level anomaly for the winter of 2013-2014. Global mean sea level is removed for the period of 1993-2016. **(d)** AVISO winter sea level anomaly and ARGO winter steric sea level anomaly over the area of 50 to 40°N, 160 to 140°W (indicated by the box shown in Figure S2a and S2c) for the period of 1993-2016 and 2001-2016, respectively.


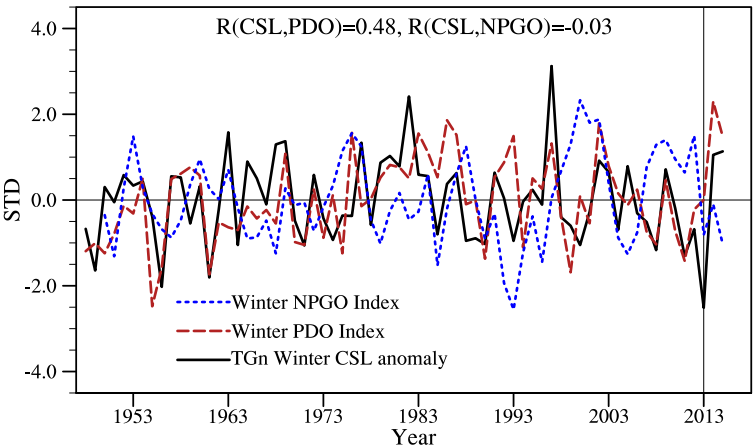


**Figure S3.** Normalized winter time series of PDO index (red-dashed line), NPGO index (blue-dashed line) and TG_N CSL anomalies (black line), respectively.


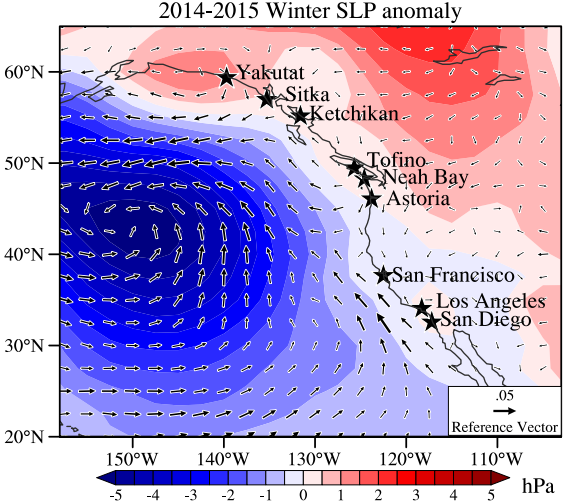


**Figure S4.** Anomalies of SLP (hPa; shading) and wind stress (N/m^2^; vector) for the winter of 2014-2015. The black stars represent the locations of TG stations.


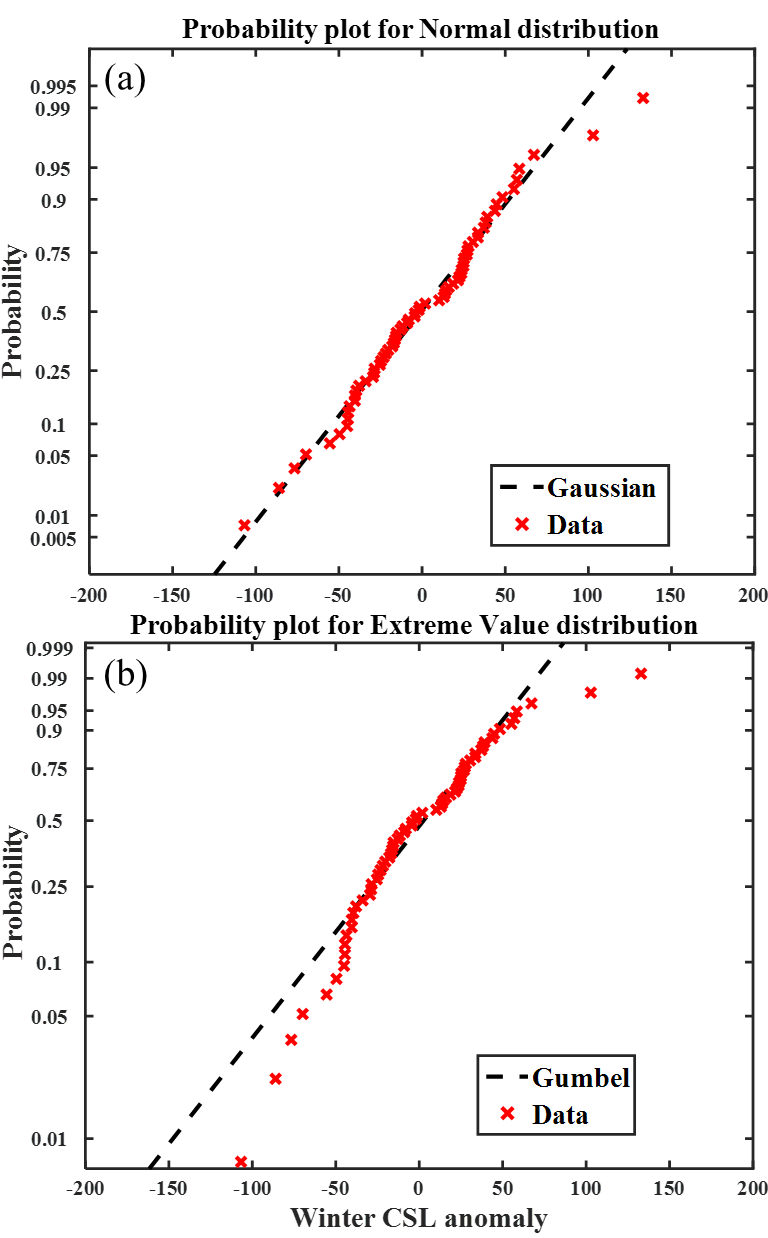


**Figure S5.** The probability plot of the winter sea level for the Gaussian distribution **(a)** and Gumbel distribution **(b)**. The more “×” that deviates from the reference (black- dashed) line, the more the distribution of data obeys a larger deviation from the assigned distribution.
